# The effect of the number of active electrode poles during tined lead placement on long‐term efficacy of sacral neuromodulation in patients with faecal incontinence

**DOI:** 10.1111/codi.15223

**Published:** 2020-07-22

**Authors:** R. Assmann, S. O. Breukink, S. A. P. Caubergh, L. P. S. Stassen, S. M. J. van Kuijk, J. Melenhorst

**Affiliations:** ^1^ Department of Surgery and Colorectal Surgery Academic Hospital Maastricht Maastricht University Medical Center Maastricht The Netherlands; ^2^ Maastricht University Maastricht The Netherlands; ^3^ Department of Clinical Epidemiology and Medical Technology Assessment Maastricht University Medical Centre Maastricht The Netherlands

**Keywords:** Sacral neuromodulation, faecal incontinence, tined lead placement, surgery

## Abstract

**Aim:**

There is an ongoing debate as to whether or not the efficacy of sacral neuromodulation (SNM) is optimized by maximizing the total number of active electrode poles (AEPs) during lead placement because there are more programming options. However, this is at the cost of increased operating time. The aims of this study were to establish if a higher number of AEPs improves SNM efficacy during the trial period and after permanent implantable pulse generator (IPG) placement and if there is there a correlation between number of AEPs and battery life of the first placed IPG.

**Method:**

This was a single centre retrospective cohort study of new patients with faecal incontinence who underwent SNM between 2000 and 2018. Exclusion criteria were sphincter defect > 30%, rectocele/enterocele Grade 3 or higher and incomplete records.

**Results:**

In all, 288/456 (63%) patients (women 91%; mean age 58.5 ± 11.7 years) were eligible for analysis. The number of AEPs during lead placement was two (*n* = 42, 14.5%), three (*n* = 82, 28.5%) and four (*n* = 164, 57%). There was no association between the number of AEPs during tined lead placement and long‐term efficacy. Neither the success rate of the trial phase nor the battery life after first placed IPG was influenced by the number of AEPs.

**Conclusion:**

In this study, the number of AEPs does not seem to influence long‐term efficacy of SNM success rate during the trial phase or the battery life of the first placed IPG. However, we also suggest that at the very least there should be two AEPs at lead placement.

**What does this paper add to the literature?**

This paper shows that the number of active electrode poles (AEPs) during tined lead placement does not influence the efficacy of sacral neuromodulation. This contradicts other studies which have reported that more AEPs optimize treatment.

## Introduction

Sacral neuromodulation (SNM) is an established treatment for faecal incontinence (FI) with a sustained efficacy between 47.7% and 52.7% [1, 2, 3, 4]. In order to improve long‐term efficacy, Dudding *et al*. [5] have suggested altering stimulation parameters whilst others have increased the number of active electrode poles (AEPs) during tined lead placement (TLP) [6]. An advantage of a greater number of AEPs is that there are more programming options. Decreasing the stimulation amplitude improves the battery life of the implantable pulse generator (IPG) and reduces the frequency and costs of battery changes. Repositioning of the lead during TLP to reach the maximum number of AEPs can be time consuming and not always successful.

There are limited data relating the number of AEPs during initial lead placement to long‐term SNM efficacy. Duelund‐Jakobsen *et al*. [7] reported that the total number of AEPs did not affect the Wexner incontinence or Visual Analogue Scale scores (influence of incontinence on daily life) after permanent SNM implant. A study assessing urinary incontinence showed that the number of AEPs did not influence successful transition to implantation of the IPG, the need for reoperation or loss of efficacy [8].

The primary goal of this retrospective study was to assess the association between the number of AEPs during lead placement and the long‐term efficacy of SNM in patients with FI. Secondary goals were to evaluate the association between the number of AEPs during lead placement and success rate during the trial phase, and to study the association between the number of AEPs and battery life of the first placed IPG.

## Method

A retrospective analysis was performed using patient records and diaries completed by patients with FI undergoing SNM between 2000 and 2018 in our hospital. Inclusion criteria were a minimum of three episodes of FI per week and failed conservative management, including diet and fluid advice, medication, biofeedback therapy and/or colonic irrigation [9]. Exclusion criteria were a sphincter defect > 30% and rectocele/enterocele Grade 3 or higher. Permanent implants used bone and fascial anchored leads before the introduction of TLP in 2003. This cohort includes patient results reported in previous communications [1, 10, 11].

Ethical approval for this study was provided by the Medical Ethical Committee aZM/UM. The STROBE guidelines have been complied with for reporting of this cohort study.

### Registration of data

Patients kept bowel diaries for 3 weeks in which they recorded daily the frequency of evacuations, episodes of FI and time (minutes) they were able to defer defaecation. They completed the diary entries at baseline, during trial screening, and at 1, 3, 6 and 12 months after IPG placement. After 1 year, patients completed an annual bowel diary. Patients attended outpatients at the above intervals.

Until 2003, permanent leads were implanted in an open procedure under general anaesthesia and electrode position was assessed using a motor response. When TLP became available, patients were offered the choice of local or general anaesthesia. It should be noted that two different IPGs were implanted in patients: the InterStim I and the InterStim II (Medtronic, Minneapolis, Minnesota, USA).

Patients who entered the trial phase but did not have a successful response (defined as 50% decrease in FI episodes) were excluded from the analysis and were not followed up. Demographic data were used to assess baseline characteristics.

### Outcome measures

The primary outcome measure was the number of FI episodes compared to baseline. These data were drawn from bowel movement diaries or the patients' files.

The secondary outcome measures were (i) the success rate during the trial phase and (ii) the battery life of the first placed IPG (time to IPG replacement or, if the IPG had not as yet been replaced, the time from first placed IPG to date of last follow‐up).

### Statistical analysis

**Figure 1:**
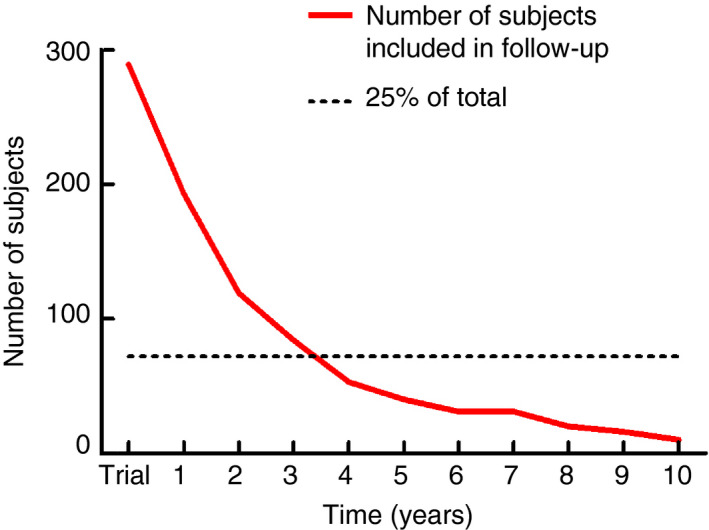
Number of subjects for whom data were available at each point of follow‐up.

Baseline characteristics were described using mean ± standard deviation or count and percentage. Differences between groups defined by the number of AEPs were tested using one‐way analysis of variance (ANOVA) for continuous variables and Pearson's chi‐squared test for categorical variables at each follow‐up moment. Since samples could differ substantially between follow‐up points, we chose not to perform longitudinal analysis. We decided to analyse only those follow‐up points at which a minimum of 25% of the cohort was observed (Fig. 1).

Battery life was analysed using the Kaplan–Meier estimator. Groups were compared using the log‐rank test. All statistical analyses were performed using ibm spss Statistics 25 (IBM, Armonk, New York, USA). *P* values ≤ 0.05 were considered statistically significant.

## Results

### Patient characteristics

In all, 456 new patients with FI who underwent lead placement between 2000 and 2018 were identified. 163 (37%) were excluded due to no record of number of AEPs during TLP, lack of preoperative/postoperative diaries or files being no longer available.

**Figure 2:**
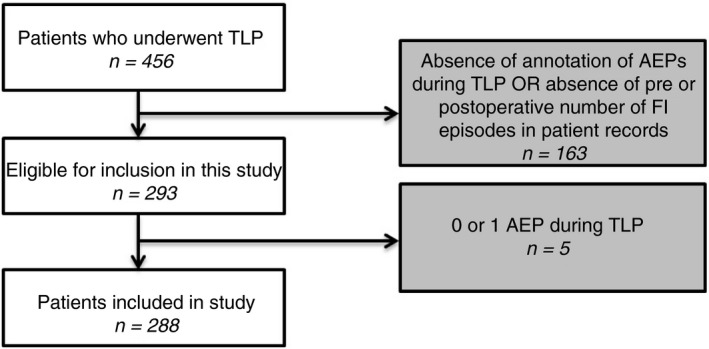
Flow chart of inclusion and exclusion of patients in this study. AEPs, active electrode poles; FI, faecal incontinence; TLP, tined lead placement.

207/293 (71%) underwent lead placement under general anaesthesia. We failed to successfully implant in 5/293 (0.02%) despite repositioning the lead (AEP 0, *n* = 2; AEP 1, *n* = 3). Both groups were deemed too small for statistical analyses and were also excluded, leaving 288 (63%) in the final analysis (Fig. 2).

### Baseline characteristics

In the cohort of 288 patients, 262 (90.9%) were women with an average age of 58.5 ± 11.7 years. Patients were divided into one of three AEP lead placement groups: two AEPs (*n* = 42), three AEPs (*n* = 82) and four AEPs (*n* = 164). The mean number of FI episodes at baseline was 36.2, 21.2 and 24.6 respectively for these groups (Table 1). The group with two AEPs showed a higher number of FI episodes per 3 weeks at baseline (*P* = 0.03).

**Table 1:** Baseline characteristics.

	Two AEPs	Three AEPs	Four AEPs	*P* value
Patients (%)	42 (14.56%)	82 (28.5%)	164 (56.9%)	–
Age (years ± SD)	60.05 ± 10.49	58.79 ± 10.59	58.25 ± 12.49	0.9110
Women (%)	40 (95.2%)	76 (92.7%)	146 (89.0%)	0.3630
FI episodes/3 weeks	36.2	21.2	24.6	0.0305*

AEPs, active electrode poles; FI, faecal incontinence.

* Significant.

### Primary outcome

**Figure 3:**
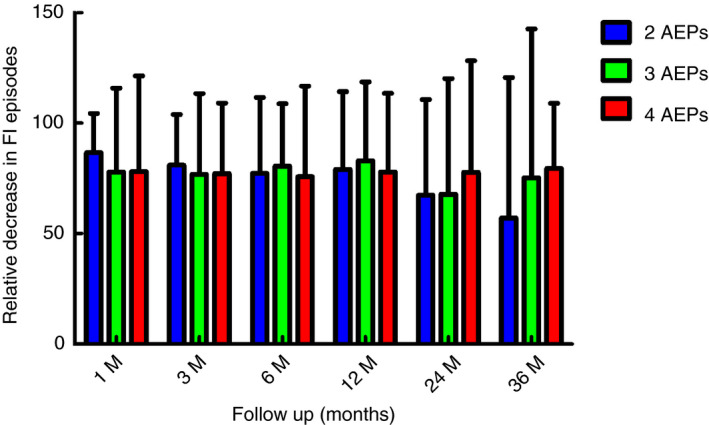
Relative decrease in number of faecal incontinence (FI) episodes at all follow‐up points compared to baseline, for groups with two active electrode poles (AEPs), three AEPs and four AEPs.

One month after placement of the IPG, a mean decrease of 76.7% in FI episodes was observed in all patients who received an IPG (*n* = 255) compared to baseline. Similar percentages were found at 3 (*n* = 215), 6 (*n* = 202), 12 (*n* = 190), 24 (*n* = 117) and 36 (*n* = 83) months of follow‐up (74.6%, 75.7%, 76.2%, 71.3%, 72.7%, respectively) (Fig. 3). One‐way ANOVA showed no differences in efficacy of SNM between the three groups (two AEPs, three AEPs and four AEPs) at 1 month (*P* = 0.524), 3 months (*P* = 0.821), 6 months (*P* = 0.717), 12 months (*P* = 0.718), 24 months (*P* = 0.581) and 36 months (*P* = 0.376) of follow‐up.

### Secondary outcome

During the trial phase, there was a mean overall decrease of 76.3% ± 32.5% in FI episodes. This figure includes patients in whom the trial phase was unsuccessful. Since baseline characteristics showed a higher number of FI episodes in the two AEP group (36.2 episodes in 3 weeks) compared to the three AEP group (21.2 episodes) and four AEP group (24.6 episodes), we assessed whether this would affect the percentage of successful trial phases between groups. The number of successful and non‐successful trials is shown in Table 2. A chi‐squared test showed no difference in success rate of the trial phase between the three groups (*P* = 0.272).

**Table 2:** Number of successful trial phases for each group.

	Non‐successful trial	Successful trial	Total
Two AEPs	4 (9.5%)	38 (90.5%)	42
Three AEPs	6 (7.3%)	76 (92.7%)	82
Four AEPs	23 (14.0%)	141 (86.0%)	164
Total	33 (11.5%)	255 (88.5%)	288

AEPs, active electrode poles.

**Figure 4:**
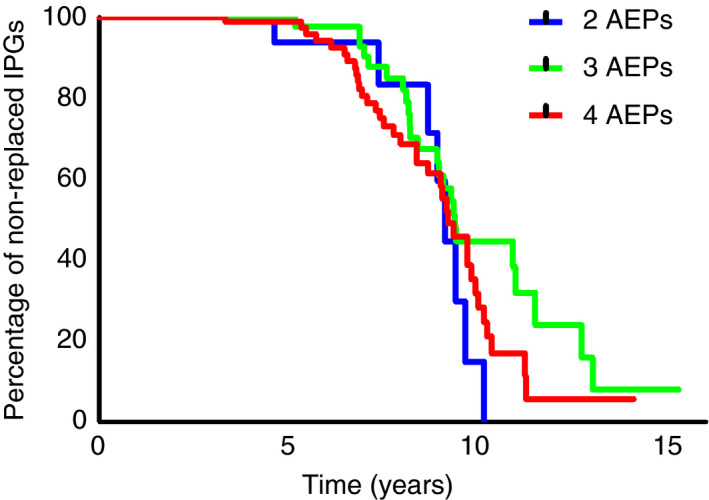
Survival of first placed implantable pulse generator (IPG) in years. AEPs, active electrode poles.

Additionally, the battery life of the first placed IPG was evaluated. Figure 4 shows the percentage of non‐replaced IPGs on the *y*‐axis over time in years on the *x*‐axis. A log‐rank test showed there was no significant difference in battery life between the three groups (*P* = 0.166).

## Discussion

In this study the number of AEPs during lead placement does not seem to have an influence on long‐term efficacy of SNM. No association was found between the number of AEPs and success rate during the trial phase, nor battery life of the first placed IPG.

At baseline, patients with two AEPs had a higher number of FI episodes compared to patients with three or four AEPs. This observation could be explained by the hypothesis that patients with only two AEPs may have more nerve damage at baseline resulting in more FI episodes. Due to this more extensive nerve damage, it may be harder to achieve motor/sensory response during lead placement. However, no evidence of this phenomenon was found in the literature. Despite the existence of a difference at baseline in FI episodes, this was not a predictor for SNM efficacy at follow‐up points.

During 3 years of follow‐up, no differences in decrease in FI episodes were found at any time point. This indicates that the number of AEPs during lead placement does not influence the long‐term efficacy of SNM on FI. This is in accordance with results published by Duelund‐Jakobsen *et al*. [7]. Moreover, Gilleran *et al*. [8] also found that the number of AEPs during lead placement did not influence the efficacy of SNM on urinary incontinence.

Our results show no correlation between the number of AEPs and success rate of the trial phase. This suggests that neither long‐term nor short‐term success rates are influenced by the number of AEPs.

Duelund‐Jakobsen *et al*. [7] showed therapeutic amplitude was significantly lower in patients with four AEPs than in patients with one, two or three AEPs, thereby implying that a decreased stimulation amplitude will improve longevity of the IPG. In contrast, we found the battery life of first placed IPG was not influenced by number of AEPs. Since we did not check the amplitude of all groups, we cannot directly refute the claim that decreased stimulation amplitude improves battery life of the IPG. However, since battery life did not depend on the number of AEPs, and previous studies have shown that < 4 AEPs results in higher amplitude, our results indirectly suggest that a higher therapeutic amplitude does not necessarily lead to decreased longevity of the IPG [7, 8].

Since 2017, use of the curved stylet is advised to position the tined lead closer to the sacral nerve root, potentially increasing the number of AEPs and programming options whilst reducing the likelihood of side effects [6]. Matzel *et* *al*. [6] state: *‘*Accepting less than four contacts close to the nerve is a compromise, which for practical reasons may be accepted'. A drawback is that the straight stylet in Medtronic's lead introduction kit has to be replaced by the curved stylet, which may lead to an increase in surgery time. Even though our study did not compare stiff stylet with curved stylet, the use of the curved stylet can be challenged, since the number of AEPs during lead placement did not influence clinical outcomes in our study or the studies performed by Duelund‐Jakobsen *et al*. and Gilleran *et al*.

A limitation of this study is that it is retrospective and there are missing data at each time point, something less likely to occur in a prospective study. This led us to perform cross‐sectional analyses, as sample size differed substantially at each point. Total follow‐up time also differed: some patients were followed up for 20 years, others for only 6 months. In order to develop conclusions based on a larger subset of our cohort, we used data of patients who had been followed up to 3 years.

A further consideration is that the analysis of the battery life of the first placed IPG does not take into account the fact that during the study two different IPGs (InterStim I and InterStim II) were implanted. One difference between the two is that the InterStim 1 battery life is twice that of the InterStim II.

SNM is a two‐staged procedure. It is at the first stage that the number of AEPs is tested. During the second stage only the energy source (either InterStim I or InterStim II) is placed. We have assumed that the number of AEPs does not differ between the two different IPGs although the large‐sized InterStim I with two AEPs and a small‐sized InterStim II with four AEPs do differ in their AEP capacity. However, assuming the same occurs in the opposite direction, the bias would be dissolved. Another limitation is the number of patients with four AEPs (*n* = 164) compared to the number of patients with two (*n* = 42) and three AEPs (*n* = 82).

In interpreting the FI, there may be a tendency for patients at baseline not to record *‘*minor soiling’ as *‘*loss of stool’, something they are more inclined to do postoperatively [7]. This could result in FI apparently less successfully treated at follow‐up points compared to baseline. However, assuming this bias did exist in our study, we would assume the effect to be similar across each of the groups.

Taking these results into consideration, we suggest SNM practitioners focus less on the number of AEPs and accept two or more AEPs in the future. Whilst we are aware of the fact that the number of AEPs is indicative for lead positioning relative to the third sacral root, this study has shown that the number of AEPs during lead placement is not beneficial for long‐term SNM efficacy, success rate during the trial phase or battery life. Correct positioning, confirmed by X‐ray and two or more AEPs, is sufficient to adequately treat FI patients with SNM. This partly agrees with earlier studies by Amend *et al*. and Williams and Siegel, who suggested accepting two or more AEPs [12, 13]. Despite this common conclusion, these two studies also suggested the optimal number of AEPs to be four, which is contrary to our findings.

In the future, it would be interesting to perform a study comparing the long‐term efficacy of SNM placed using the stiff stylet and the curved stylet.

## Conclusion

In conclusion, we observed an inverse association between number of FI episodes at baseline and number of AEPs during TLP. The number of AEPs does not seem to influence long‐term efficacy of SNM, the success of the trial phase or the battery life of the IPG. Four AEPs should always be the ultimate goal during SNM surgery but a minimum of two AEPs appears sufficient for adequate short‐ and long‐term effect and, by accepting two or more AEPs, the need for extensive repositioning would disappear, saving time in the operating room.

## Conflicts of interest

S.O. Breukink would like to declare she has received an unrestricted research grant from Medtronic. The other authors have no conflict of interest to declare.
